# Understanding adverse childhood experiences and the call for trauma-informed healthcare system in Turkey: a review

**DOI:** 10.1186/s12961-024-01137-3

**Published:** 2024-05-31

**Authors:** Nadire Gülçin Yildiz, Halide Z. Aydin, Kemal Aydin, Hatice Yildiz, Grace Sambo, Bwanalori Mwamulima, Joe Maganga Zonda, Doreen Phiri, Yohane Vincent Abero Phiri

**Affiliations:** 1 Department of Educational Sciences, Faculty of Education, Boğaziçi University, Bebek, Istanbul, 34342 Turkey; 2 Faculty of Education, Department of Guidance and Counseling, Istanbul Medipol University, Istanbul, Turkey; 3 Arnold School of Public Health, University of South Carolina, Columbia, SC USA; 4 Faculty of Economics and Administrative Sciences, Amasya University, Amasya, Turkey; 5 Health Sciences Institute, Istanbul Medipol University, Istanbul, Turkey; 6 Chang Gung Medical Education Research Centre (CG-MERC), Chang Gung Memorial Hospital, Taoyuan, Taiwan; 7 Directorate of Health and Social Services, Rumphi District Council, Rhumpi, Malawi; 8 Department of Economics, National Cheng Kung University, Tainan, Taiwan; 9 School of Nursing, College of Nursing, Taipei Medical University, Taipei City, Taiwan; 10 Department of Epidemiology and Environmental Health (EEH), University at Buffalo, Buffalo, NY USA; 11 Charis Professional and Academic Research Consultants (CPARC), C/O P.O. Box 132, Mchinji, Malawi; 12 Malawi Environmental Health Association (MEHA), P.O. Box 381, Lilongwe 3, Malawi

**Keywords:** Changing demographics, Health conditions, Risk factors, Adverse childhood experiences, Trauma informed healthcare system, Public health, Community mental health, Healthcare policies

## Abstract

Over the past four decades, research has underscored the significance of approaching and preventing trauma from a systemic standpoint. Trauma-informed care (TIC) methodologies offer a structure for healthcare practices, striving to convert organizations into trauma-informed systems that employ trauma-specific interventions. This review employs epidemiological and household data from Turkey to underscore the importance of integrating trauma-informed care as a means of prevention and intervention. Through a desk review, the study examines the role of adverse childhood experiences (ACEs), delving into their origin from family dynamics, migration, violence, exposure to violence, juvenile delinquency, and child maltreatment. The research highlights innovative healthcare approaches that leverage data to address complex patient health issues while considering mental health needs. In contemporary times, healthcare organizations acknowledge the value of a data-driven approach to make informed clinical decisions, enhance treatment procedures, and improve overall healthcare outcomes. The reviewed research and empirical data furnish proof of the importance of effective and efficient treatment methods that prioritize trauma prevention and treatment, integrating the role of ACEs. This paper seeks to contribute to discussions on transforming the healthcare system to meet the healthcare needs of Turkish households, all the while taking into account the evolving sociopolitical factors that shape Turkey's population characteristics.

## Background

Adverse childhood experiences (ACEs) encompass a range of traumatic events during childhood, such as abuse, neglect, or household dysfunction, which significantly impact long-term health outcomes. A trauma-informed healthcare system recognizes the prevalence and effects of ACEs, guiding healthcare professionals to approach patient care with sensitivity, understanding, and a focus on creating a supportive environment that addresses the potential impact of past traumas [70]. Literature indicates that exposure to trauma during childhood, either as direct victims or indirect witnesses, leads to social and emotional injuries that may manifest academically and behaviorally [65]. Additionally, these children might display poor academic achievement, maladaptive behavior, attention deficits, and an increase in both absenteeism and dropout rates [65, 68, 69]. The Substance Abuse and Mental Health Services Administration [81] outlines four characteristic features of a trauma-informed health system, which involve (a) acknowledging the impact of trauma and its potential for recovery, (b) identifying signs and symptoms of trauma in system members, (c) incorporating knowledge about trauma into policies, procedures, and practices, and (d) actively avoiding re-traumatization [68].

Data released by the Turkish Statistical Institute [89] indicates that the average family structure and size in the country, along with the prevalence of marital status, are undergoing continuous changes, significantly impacting the mental health of children. Furthermore, as per the UN Refugee Agency (UNHCR) Fact Sheet from September 2020, Turkey hosts the world's largest population of Syrian refugees, numbering 3.6 million, all of whom are under temporary protection. Nearly half of this population, specifically 1.6 million individuals, comprises children aged between 0 and 18 years old. Official documents reveal that over one million refugees fall within the school-going age category, with an average school enrollment rate of 39% [98]. Literature establishes a foundational basis to support the argument for integrating a trauma-informed healthcare system into Turkey's public health policies [88, 95, 96]. This review and analysis, therefore, aims to explore this perspective.

Socioeconomic factors, along with household and neighborhood characteristics, play a crucial role in shaping children's well-being. They impact learning experiences, attitudes toward schooling, general mental health, safety, and overall welfare. ACEs, categorized according to the ACE study, fall into three groups: (a) abuse (emotional, physical, sexual); (b) household challenges (intimate partner violence, substance abuse, mental illness, separation, divorce, incarcerated household member); and (c) neglect (emotional and physical). These factors pose risks to children's social-emotional, cognitive, physical, and behavioral development [25]. As per the Address-Based Population Registration System (ABPRS) findings, Turkey's population stood at 83,614,362 by the close of 2020, with 22,750,657 being children. Among them, 51.3% were boys, and 48.7% were girls. As per the United Nations' definition, encompassing individuals aged 0–17 as children, minors constituted 48.5% of the total population in 1970, a figure that declined to 41.8% by 1990 and further decreased to 27.2% by 2020. Projections indicate that this ratio is expected to decrease to 26.6% by 2025, 25.6% by 2030, 23.3% by 2040, 20.4% by 2060, and 19.0% by 2080 [90].

Turkey faces unique healthcare challenges due to its demographic characteristics. As of May 2018, 45.8% of Syrian refugees in Turkey are under 18 years old, according to data from the Interior Ministry’s General Directorate of Migration Management. Additionally, according to United Nations [97]  International Migration Statistics , the immigrant population in Turkey increased by 17.2%, totaling 677,000 compared to the previous year. With these shifting demographics, Turkey's healthcare system requires significant transformations, addressing factors such as birth and mortality rates, family structure, marriage figures, health issues, housing, and migration rates (both rural to urban and transnational migration, as well as birth rates among immigrants).

Despite the achievements of the Health Transformation Program (HTP) in overhauling the healthcare system, including efforts to ensure universal access, upgrade infrastructure, and improve specialized care for conditions like cardiovascular diseases and diabetes, there is still much work to be done [10, 102, 104]. Ensuring the ongoing success of Turkey's HTP is vital, and addressing potential threats to the healthcare system and its progress is crucial [4]. ACEs have the potential to overwhelm healthcare systems as traumatic events during childhood may affect long term health outcomes [50]. Addressing ACEs through prevention measures and support can improve health outcomes and potentially lessen the strain on the healthcare system. The influx of immigrants due to political instability in Turkey's neighboring regions, coupled with socioeconomic and political challenges in the area, poses risks to the mental health and well-being of individuals, whether they are displaced voluntarily or involuntarily [35]. These events are a recipe for increased ACEs and a flop in the healthcare system if not carefully addressed.

Early identification of ACEs' influence on developmental and adult psychopathology can mitigate their effects [21, 24, 35, 51, 62, 72, 75, 76]. Current demographic trends and evidence-based outcome studies advocate for healthcare policy transformations centered on ACEs [50]. Considering Turkey's evolving demographics [93], the undeniable role of ACEs in the physical and mental health of future population cohorts underscores the need for trauma-informed healthcare [25]. Therefore, this desk review examines the role of various factors linked to ACEs and the effects of ACEs on the healthcare system, using epidemiological studies and demographic databases. Additionally, we highlight the necessity for a trauma-informed healthcare system to guarantee the ongoing success of the Turkish Health Transformation Program.

## The origin of ACE factors

The original CDC-Kaiser Permanente Adverse Childhood Experiences Study (ACEs) was conducted at Kaiser Permanente from 1995 to 1997, encompassing two waves of data collection. It stands as one of the largest inquiries into childhood abuse, neglect, household challenges, and their correlation with later-life health and well-being [13]. In this study, over 17,000 Health Maintenance Organization members from Southern California, who underwent physical exams, were surveyed confidentially about their childhood experiences, current health status, and other behaviors. Felitti et al.’s [25] article, titled “Relationship of Childhood Abuse and Household Dysfunction to Many of the Leading Causes of Death in Adults,” is a highly cited work emphasizing the significant impact of specific childhood adversities on both physical and behavioral health outcomes.

## Factors linked to adverse childhood experiences (ACEs) in Turkey

### Changing family dynamics

Family dynamics in Turkey are undergoing significant changes. Parental verbal affection and aggression during childhood have distinct impacts on psychiatric symptoms and well-being in young adulthood [63]. In 2019, 9.2% of Turkish households were comprised of single parents and children, posing a risk factor for childhood adversity. Detailed analysis revealed that 2.0% consisted of fathers and children, while 7.2% were mothers and children. According to the Address Based Population Registration System (ABPRS), among 22,876,798 children, 268,843 lost their fathers, 81,239 lost their mothers, and 4649 lost both parents [91].

Divorce significantly impacts children's social and emotional well-being, relationship dynamics, mental health, and overall coping mechanisms. In 2020, 35.3% of divorces occurred within the first five years of marriage, while 20.7% took place between 6 and 10 years into marriage [90]. During that year, 135,022 couples divorced, affecting 124,742 children based on custody arrangements. Notably, the majority of children (75.8%) were granted custody to their mothers, with the remaining 24.2% to fathers. Children under 18, as well as those over 18 with disabilities, were included in custody decisions resulting from divorce. Children of divorced, separated, or deceased parents face heightened risks of childhood adversity [1, 25].

Divorce is widely recognized as a factor contributing to child poverty, which, in turn, significantly contributes to ACEs. Child poverty, identified as a major risk factor, has detrimental effects on mental health [25]. In 2018, the poverty rate for single parents with at least one child was 17.1% [91]. The proportion of households led by single parents with at least one resident child was 8.9% in Turkey during the same year. Upon closer examination, 1.9% of households consisted of single fathers with at least one child, while 7% were single mothers with at least one resident child [91].

### Migration

Migration statistics from both TurkStat and the UNHCR reveal that 3.6 million Syrian refugees have arrived in Turkey in significant waves in recent years. The Syrian Civil War, impacting the region south of Turkey's border, has forced relocation, leading to severe social, emotional, and physical challenges for the refugees. This crisis, stemming from forced migration, demands comprehensive action to address entrenched inequities and inter-generational trauma resulting from systemic discrimination or racism upon arrival. Implementing transformative changes at a societal level is essential. Including more robust early childhood programs is crucial in mitigating the adverse effects experienced at a young age, which can disrupt development and impact both the nervous system and physiology [55].

Forced migration has been extensively studied for its impact on mental health, affecting coping mechanisms, stress management systems, and related physiological defenses, potentially leading to harmful consequences [38, 54, 66]. Oklahoma, a southern US state, has a history marked by trauma and adversity, exemplified by events such as the Tulsa Massacre of 1921 and the forced relocation of Native American tribes [23, 52]. Exploring the biology of adversity and resilience has the potential to transform both children's and adults' physical and mental health practices [75, 76]. The interaction of genes with environmental factors determines coping mechanisms over time in the context of the biology of adversity and resilience [9]. Another study links early childhood adversity, toxic stress, and the impacts of racism, illustrating the extensive effects of such a dangerous web on health [75, 76]. In essence, the mind is intricately connected to the body, with early childhood development profoundly influencing lifetime health [55].

In Turkey, approximately one-fourth (26%) of Syrian refugee children have reported experiencing cruelty or torture related to flight or migration. Forced removal from one's homeland can pose a developmental risk factor, contributing to mental health issues and psychopathology, particularly observed among Syrian children and adolescents in Turkey [35]. Additionally, 70% of Syrian children and adolescents in Turkey have witnessed explosions or armed conflict, while 68% have observed combat or explosions [14, 35].

### Juvenile delinquency

Between 2015 and 2019, 511,247 police incidents involved children, with 46.1% identified as victims, 32.9% suspected of committing a crime, 15.1% providing information, and 3.4% reported missing. Of the 235,931 children identified as victims, 87.5% were victims of crime, while 12.5% were involved in incidents requiring follow-up. Notably, 53.8% of crime victims were boys [95]. Moreover, the number of Turkish children involved in crime doubled over the last decade, with 601,754 incidents recorded in 2022, marking a 20.5% increase from 2021. Additionally, 43.1% of juveniles brought to correctional offices had themselves been victimized [95]. Interestingly, out of the 206,498 children who came to security units as victims, 57.6% were victims of injury, 15.2% of sexual crimes, and 11.0% of crimes against family order. Additionally, 3.5% of the victims were reported to have made threats, 2.7% had "inhibited someone's personal freedom," and 2.1% were accused of theft (TurkStat Statistics on Family 2019). Previous studies have shown that ACEs are particularly prevalent among children involved in the justice system [5, 29, 56]. Experiencing ACEs is linked to a dose–effect relationship with chronic physical health, mental health, and behavioral outcomes among juveniles [5]. This suggests that a higher number of ACEs is associated with an increased risk of adverse outcomes in various aspects of the well-being of justice-involved children.

### Child maltreatment and violence

It is a well-established fact that violence and exposure to violence significantly impact the lives of teenagers. According to a 2019 youth risk survey by the CDC [84] in the United States, 1 in 7 youths have experienced two or more types of violence, with nearly 44% dealing with at least one form of violence. The experience of violence is associated with a minimum of 16 health conditions and risky behaviors. However, it is crucial to recognize that violence is preventable through adherence to health-promoting public health policies, enabling young people to grow up in environments free from violence both at home and in their surroundings.

The repercussions of violence are far-reaching, limiting opportunities and potentially leading to emotional and physical health issues that impact longevity. Teenagers aged 14 to 18 often face various forms of violence, including physical fights, bullying, sexual assault, and dating violence. Regrettably, homicide ranks as the third leading cause of death among teens, with females more likely than males to experience three or more forms of violence. The COVID-19 pandemic has heightened the risk of cyberbullying and threats for some teens. The consequences of violence extend to various aspects of teens’ lives, affecting school attendance and access to community support services. Teens exposed to violence often exhibit health conditions and risky behaviors such as missing school due to safety concerns, lower academic performance, weapon carrying, suicidal thoughts or behavior, engagement in risky sexual behavior, overweight or obesity, feelings of sadness or hopelessness, and substance use [84].

Notably, when teens experience violence, it has a profound impact on their mental health, influencing school attendance due to safety concerns (with percentages increasing from 3.8% for those with no violence experience to 34.1% for those experiencing three or more forms of violence). Similarly, rates of suicidal ideation escalate with the number of violence forms experienced, emphasizing the urgent need for comprehensive intervention and prevention strategies [84].

Hays-Grudo and Morris [37] emphasized the detrimental impact of violence on teenagers' development and their current and future health. Preventing violence is crucial for supporting the well-being of adolescents and adults alike. Teenagers, whose brains are still developing, may face negative consequences from experiencing violence, including impaired decision-making, learning challenges, reduced connections with peers and adults, difficulty coping with stress, and an increased likelihood of engaging in violence. Furthermore, exposure to violence during adolescence can also affect physical health, potentially leading to the development of cancer, heart disease, or other health issues in adulthood. By proactively preventing violence, a conducive environment is created for teenagers to grow into healthy adults. Disturbingly, Listenbee et al. [46] documented that 46 million children witness violence, crime, and various forms of abuse annually in America.

### ACEs impact on lifelong well-being

ACEs encompass three main categories: (a) abuse (emotional, physical, sexual); (b) household challenges (intimate partner violence, substance abuse, mental illness, separation, divorce, incarcerated household member); and (c) neglect (emotional and physical). These factors pose risks across social-emotional, cognitive, physical, and behavioral domains, which can be preventable. The CDC [13] Kaiser ACE study is the largest investigation into childhood abuse, neglect, and household challenges, examining their impact on later-life health and well-being. The relationship between childhood adversity and household dysfunction underlies some of the leading causes of death in adults. While prior research focused on specific traumas or parental variables, recent studies integrate multiple adversities, utilizing ACEs factors to assess the effects of exposure to various forms of abuse, neglect, family dysfunction, and household challenges [5, 39, 46]. Outcomes measured include prevalent health diagnoses, general health assessments (e.g., hypertension), stress-related bodily reactions (e.g., elevated cortisol levels), behavioral issues, cognitive functioning (e.g., growth and learning), and executive functioning aspects (e.g., decision-making, attention, memory, and self-regulation skills).

ACEs elevate health risks, leading to lifelong social and economic challenges. In 2017, ACEs resulted in around $5.2 billion in direct medical expenses and lost productivity among Tennessee adults, primarily due to worker absenteeism [49, 58]. ACEs are linked to risky behaviors and adverse health outcomes, contributing to higher medical costs and employee absenteeism related to health issues [49]. Vigo et al. [100] argue that the global burden of mental illness is underestimated when relying solely on reports, documents, and statistics. They predict that mental illness contributes to 32.4% of years lived with disability (YLDs) worldwide. The average length of stay in hospitals for mental and behavioral disorders increased about four times more than any other category from 2013–2016, as reported by the Turkish Ministry of Health [88], based on the General Directorate of Health Services Diagnosis-Related Groups Database.

We previously demonstrated the link between ACEs and factors such as migration, violence, family dynamics and juvenile delinquency. The CDC [13] states that populations with a history of ACEs often experience chronic health issues in adulthood, leading to higher medical expenses. A study conducted by Bethell et al. [7], utilizing data from the National Survey of Children's Health from 2011 to 2012, examined the impact of ACEs on children and teenagers aged 6 to 17. The results from the study revealed that 48% of parents reported that their children had experienced at least one of the nine ACEs, with 22.6% reporting two or more. Furthermore, children with two or more ACEs were nearly three times more likely to repeat a grade compared to those with no ACEs, even when accounting for demographic and health factors. The dose–response effect showed that children with multiple ACEs were more likely to face social and educational challenges. The findings demonstrated the need for healthcare practices to address the long-term effects of ACEs during childhood. Hays-Grudo and Morris [37] recommend using evidence-based strategies to protect children from ACEs and mitigate any negative effects as they grow into adulthood.

After nearly three decades of research, it appears there is much to be learned from ACEs today in terms of the rollout of public health initiatives and policies, with a strong indication that healthcare must thus be informed on the role of trauma. Exposure to violence within the community is a form of traumatic experience according to the research on ACEs [13]. Studies have shown that experiencing trauma significantly increases the likelihood of developing mental health issues, difficulties in social relationships and behavior, physical illnesses, and poor academic performance [32]. Trauma can lead to academic struggles due to lack of focus, problems with processing new information, and increased absenteeism. Creating a supportive and positive school environment can help mitigate these effects [20]. It is important to help students succeed in the face of challenges and adversity [83]. A trauma-informed school is one where all students feel safe and supported [17]. Research has shown that mentoring programs can reduce reoffending and delinquent behavior among at-risk youth [41, 86]. Exposure to one form of violence increases the risk of experiencing other forms of violence [53]. Finkelhor [26] found that assault in 2015 was associated with a higher likelihood of sexual victimization and caregiver maltreatment. Additionally, one in six children experienced multiple types of victimization, highlighting the impact of trauma. The effects of trauma can be passed down to future generations and can affect the brain and mind at a cellular and molecular level [6, 8, 34, 60, 61, 99].

The connection between adverse childhood experiences (ACEs) and health outcomes has also been explored through biological and neurological pathways. Boyce et al. [9] emphasize the association between ACEs and health outcomes through epigenetic mechanisms. Exposure to ACEs in childhood chemically induces stress, potentially shortening gene alleles, which are then linked to various health issues such as cancer, dementia, diabetes, and heart disease later in life. It is undeniable that toxic stress alters human biological chemistry, giving rise to developmental trauma that, in turn, modifies the brain's neural networking system in both structure and functional operation. When stress is intense, prolonged, and frequent, it becomes toxic, triggering a persistent fear response. Teicher and Samson [82] argue that the nature and timing of adversity are crucial, as being nurtured up to or by the age of four predicts brain volume at age fourteen. Highlighting the impact of adverse childhood experiences (ACEs) on healthcare systems, the CDC (2016) underscores the necessity of promoting social norms that safeguard against violence and adversity. This involves educating children and youth on skills to effectively manage stress and intervening to reduce both immediate and long-term harms. The biological and neuropsychological consequences of ACEs have also been linked to physical abuse in children [44]. According to Teicher and Samson [82], certain forms of childhood physical abuse, including sexual abuse, impact the development of the cerebral cortex, thereby influencing brain function. Interestingly, sexual abuse affects the visual cortex responsible for facial recognition. In the United States, Bessel (2014) asserts that ACEs related to child maltreatment constitute the most expensive public health issue. Just one ACE has the potential to shorten a person's life by an average of up to 20 years. Fisher [28] points out that ACEs contribute to a decrease in overall life expectancy, and individuals who experienced one ACE during childhood are 46 times more likely to develop drug dependencies, particularly males.

### The need for trauma informed healthcare system in Turkey

Over the last three decades, the idea of trauma-informed care has developed, incorporating insights from different schools of thought and innovations. Currently, it is applied in a variety of settings, including mental health facilities, substance abuse rehabilitation centers, child welfare systems, schools, and criminal justice institutions [101]. Despite its broad application, trauma-informed care does not adhere to a one-size-fits-all approach. Interventions need to be customized based on each client's distinct situation, considering factors such as gender and the specific type of trauma encountered, ACEs included [43]. This summative review demonstrates that experiencing trauma, especially during childhood in the form of ACEs, significantly increases the chances of experiencing severe health issues throughout one's lifetime. These issues include chronic lung, heart, and liver diseases, as well as depression, sexually transmitted diseases, and the misuse of tobacco, alcohol, and illicit drugs [25, 31, 64]. Moreover, childhood trauma is linked to higher social service costs [11, 70]. In a trauma-informed health system, healthcare providers and institutions aim to comprehend the prevalence and impacts of trauma. Consequently, they incorporate trauma-sensitive practices into their care and promote a supportive and non-retraumatizing atmosphere [68]. Additionally, this entails training staff to identify and respond to trauma, adjusting communication strategies to be trauma-sensitive, and structuring services to reduce the risk of re-traumatization. Key components of this approach encompass understanding trauma's prevalence and effects, integrating trauma-sensitive practices into clinical care, fostering a safe and supportive environment, and involving patients in their care decisions [70]. Acknowledging the potential impact of past traumatic experiences on an individual's health, the trauma-informed approach transcends any specific healthcare system but can be integrated into various healthcare settings. It requires healthcare professionals to be trained in recognizing signs of trauma, adopting trauma-sensitive language and communication strategies, and cultivating a culture of empathetic understanding [70].

In a previous section of this paper, we emphasized that most improvements in the operation of the Turkish healthcare system are credited to the Turkey Health Transformation Program (HTP). To maintain the success of the HTP in Turkey, it is imperative to address potential threats to the healthcare system [4]. Health transformation programs typically strive to enhance healthcare delivery, improve patient outcomes, and address systemic challenges within the healthcare system [2, 3]. Conversely, trauma-informed healthcare is an approach that acknowledges and responds to the impact of trauma on individuals seeking healthcare services, emphasizing the creation of environments sensitive to the needs of trauma survivors. Within the framework of a health transformation program, the incorporation of trauma-informed healthcare principles has the potential to augment the overall effectiveness of the transformation. For instance, by integrating trauma-informed practices into program design, healthcare providers can establish a more supportive, patient-centered healthcare environment, and offer relevant staff training to deliver sensitive healthcare services, [70].

### Strength and limitations

This desk review utilizes existing literature to provide a comprehensive overview of the current knowledge on adverse childhood experiences (ACEs) and the importance of trauma-informed healthcare services in Turkey. It effectively combines various perspectives, research studies, and expert opinions to establish a solid understanding of the complexities surrounding ACEs and their impact on healthcare. The review also identifies areas where more research is needed and offers valuable insights for future policy development. However, it is important to recognize the limitations of relying solely on existing literature, as it may not capture recent developments or cultural nuances specific to Turkey. Additionally, the absence of primary data collection may limit the depth of understanding, particularly regarding cultural factors that influence the occurrence and management of ACEs in Turkey. Despite these limitations, this review serves as a valuable starting point for raising awareness and initiating discussions on the necessity of trauma-informed healthcare services in Turkey.

## Conclusion

This review delved into the role of ACEs, addressing debates concerning (a) shifting family dynamics, (b) migration, violence, and exposure to violence, (c) the juvenile justice system in Turkey, and (d) child maltreatment and violence affecting the lives of teenagers. Migration, both on a national scale (from rural to urban areas) and a transnational scale (particularly from Syria to Turkey), exerts a significant influence in these transformations, posing challenges to the intricate sociodemographic landscape. The aim is to stimulate discussions on revising the healthcare system to effectively address the evolving needs of the Turkish population, considering the impact of ACEs on healthcare expenditure.

The Turkish healthcare system has undergone significant changes in recent years, demonstrating resilience amidst challenges like the COVID-19 pandemic, conflicts in Ukraine and Russia, the Israeli-Palestinian conflict in the Middle East, and an earthquake in southern Turkey. Working with ACEs enables the integration of trauma-informed healthcare, serving as a community-based preventive public health strategy that can reduce medical expenses while enhancing resilience in children and young adults confronting various emerging challenges. Changing demographics are impacting healthcare systems worldwide, including in Turkey, where dramatic shifts in birth rates, mortality rates, and household variables have occurred, resulting in the emergence of new societal needs. Additionally, factors such as migration, both at the national level (from rural to urban areas) and the transnational level (Syrians seeking refuge in Turkey), contribute to an increasingly complex sociodemographic landscape. Moreover, the global COVID-19 pandemic has significantly influenced demographic trends. Given these shifts and more, initiating discussions on revising the country's healthcare system to meet the population's evolving needs has become imperative.

Despite the success of HTP dating 2003, change in the Turkish healthcare is inevitable. Yilmaz [105], in a book titled “The Politics of Healthcare Reform in Turkey”, demonstrates that healthcare initiatives must align with everyone's basic citizenship rights, in accordance with the goals of community public health policies. Demographic variables associated with changing family dynamics, household factors, and migration underscore the importance of the Trauma-Informed Healthcare System in Turkey as these worsen the prevalence of ACEs. The recent health crisis of COVID-19 in Turkey, coupled with the aftermath of a powerful earthquake almost a year ago, has left 9.1 million people in need of support, with the death toll surpassing 50,000 and impacting 11 provinces [94]. Therefore, a sustained systemic change is necessary in the Turkish healthcare system to address the needs of the population resulting from these complex range of incidents. Atun [4] argues that for Turkey to transition from a middle-income to a high-income status, the country should base its economy on data, with the health system playing a major role.

## Data Availability

The data supporting this scoping review's findings is available from the corresponding author upon a reasonable request. Furthermore, the references mentioned in this paper are accessible through the sources provided.
